# Linking perceived film and television narrative rhythm with audience emotional immersion: the mediating paths of attention allocation and narrative transportation

**DOI:** 10.3389/fpsyg.2026.1971219

**Published:** 2026-10-28

**Authors:** Xiaoshi Lv

**Affiliations:** College of Media, Harbin Normal University, Harbin, Heilongjiang, China

**Keywords:** attention allocation, chain mediation, emotional immersion, film and television narrative rhythm, media psychology, narrative transportation

## Abstract

**Introduction:**

With the trend of fragmented and mobile film and television viewing, narrative rhythm has become an important factor closely related to audience experience. Drawing upon foundational cognitive attentional principles and narrative transportation theory, this study examines the associative mechanisms and structural paths linking perceived film and television narrative rhythm (PNR) with audience emotional immersion (EI).

**Methods:**

Based on cross-sectional survey data collected from 1,074 participants, the study employed Structural Equation Modeling (SEM) and Bootstrap resampling procedures (5,000 resamples) to evaluate direct and indirect associations among variables, while controlling for trait empathy and demographic characteristics.

**Results:**

Perceived narrative rhythm was significantly and positively associated with emotional immersion. Attention allocation (AA) and narrative transportation (NT) each demonstrated significant independent indirect associations between perceived rhythm and emotional immersion, while jointly constituting a significant chain mediation pathway: perceived narrative rhythm → attention allocation → narrative transportation → emotional immersion. Following the inclusion of these mediating variables, the residual direct association between perceived rhythm and emotional immersion was no longer significant. This associative pattern demonstrated consistency across gender, age, and viewing device subgroups.

**Discussion:**

The findings illustrate the sequential cognitive and experiential patterns whereby audiences transition from cognitive resource allocation to story space involvement during media processing. This study provides an empirical foundation from a psychological perspective for understanding audience media engagement and emotional immersion in the mobile digital era.

## 1 Introduction

### 1.1 Research background

The popularity of the mobile internet and streaming platforms has deeply integrated film and television content into multi-dimensional device scenarios such as mobile terminals, directly driving the evolution of audience viewing behavior toward mobility and fragmentation (Appel and Mengelkamp, 2022; Cao et al., 2021). In a competitive platform environment, film and television works need to present character relationships, plot conflicts, and narrative information within a limited time, making film and television narrative rhythm an important issue in film and television creation and audience research (Cutting et al., 2010).

Previous studies have highlighted that formal structural features, such as editing pace, event density, and shot length, heavily influence viewer cognition (Zou et al., 2025; Cutting et al., 2010). Building upon these objective textual elements, this study introduces and focuses on an extended subjective construct: perceived film and television narrative rhythm. This subjective evaluation involves not only the speed of plot advancement but also the audience’s cohesive perception of event density, shot switching, dialog variations, conflict frequency, and the alternation of climax and soothing segments. Web series and micro-dramas often use content organization methods such as front-loaded conflicts, suspense settings, and plot reversals that integrate high audiovisual intensity with narrative appeal (Gan et al., 2023). Therefore, narrative rhythm cannot be simply equated with “fast” or “slow”; attention should also be paid to information density, audiovisual tension, and rhythm fluctuations.

Film and television rhythm has manifestations at both the textual and audiovisual levels, but audiences’ judgments of rhythm are influenced by genre familiarity, viewing habits, media experience, and personal preferences. This study does not directly measure the objective shot length or number of plots in a work, but rather examines the subjective judgments formed by audiences in specific viewing contexts.

During the viewing process, the audience’s attention may be disturbed by environmental sounds, message notifications, social interactions, and other media activities. Concentration of attention is not equivalent to entering the story world. Audiences may understand the plot through sustained attention, or they may form narrative transportation through character identification and situational imagination. Emotional immersion is reflected in the emotional connection between the audience and the characters, events, and story atmosphere, including emotional resonance, physiological responses, and emotional continuation after viewing.

Existing studies have, respectively, discussed media information processing, narrative engagement, narrative transportation, and entertainment experience, but integrated analyses of the statistical connections among perceived film and television narrative rhythm, attention allocation, narrative transportation, and emotional immersion are still insufficient (Kubrak and Starostina, 2025; Liu and Deng, 2025; Wang and Tang, 2021; Zhou et al., 2026). This study takes a film or television work recently viewed or that left a deep impression on the respondents as the recall object to examine the relationship between perceived film and television narrative rhythm and audience emotional immersion, and analyzes the indirect statistical paths presented by attention allocation and narrative transportation therein.

### 1.2 Research questions

This study revolves around the following questions:

*RQ1*: Is there a correlation between perceived film and television narrative rhythm and audience emotional immersion?

*RQ2*: What kinds of correlations do perceived film and television narrative rhythm present with attention allocation and narrative transportation, respectively?

*RQ3*: Does attention allocation present an indirect association between perceived film and television narrative rhythm and audience emotional immersion?

*RQ4*: Does narrative transportation present an indirect association between perceived film and television narrative rhythm and audience emotional immersion?

*RQ5*: Do attention allocation and narrative transportation constitute a chain indirect statistical path of “perceived film and television narrative rhythm — attention allocation — narrative transportation — audience emotional immersion”?

*RQ6*: What role do the audience’s individual difference characteristics (such as trait empathy, demographic variables, etc.) play in the aforementioned variable associations?

### 1.3 Research significance

This study discusses film and television narrative rhythm from the perspective of audience subjective perception, distinguishing between the objective formal features of the work and the audience’s rhythm judgment, which helps to connect film and television text research with audience psychological measurement.

The study measures attention allocation, narrative transportation, and emotional immersion as different constructs. Attention allocation focuses on the concentration of cognitive resources, narrative transportation focuses on psychological entry into the story world, and emotional immersion focuses on the audience’s emotional experience. Distinguishing the three helps reduce conceptual confusion and test their statistical positions in the theoretical model.

Combining the limited capacity information processing perspective and narrative transportation theory, this study analyzes the correlation structure among cognitive attention, story experience, and emotional immersion. Because a cross-sectional survey is used, the research results are mainly used to integrate variable relationships and test the mediation structure model, providing references for subsequent experimental and longitudinal studies.

At the practical level, the research results can provide references for film and television content evaluation, audience analysis, and content testing. The study focuses on the coordination among narrative advancement, amount of information, audiovisual tension, and rhythm fluctuations, rather than simply increasing plot speed or conflict density. The relevant results should be comprehensively interpreted in combination with platform data, interview materials, and actual viewing behaviors.

## 2 Literature review and theoretical framework

### 2.1 Definition of core concepts

#### 2.1.1 Perceived film and television narrative rhythm (PNR)

Film and television narrative rhythm is the way a work organizes story information and audiovisual materials in the time dimension, involving plot advancement speed, event density, shot transitions, dialog density, music changes, and the configuration of climax and soothing segments. The film form study by Bordwell et al. (2008) specifically addresses objective formal components, pointing out that actual narrative time, shot organization, and plot structure jointly participate in the audience’s understanding of the film’s progress.

Perceived film and television narrative rhythm emphasizes the audience’s subjective judgment of the above features. Lang’s (2000) Limited Capacity Model of Mediated Message Processing (LC4MP) does not explicitly center on “narrative rhythm” per se, but instead provides a foundational cognitive mechanism: it indicates that the cognitive resources individuals use to process media information are limited, and there is a connection between formal information structure and resource allocation (Fisher et al., 2018; Lang, 2000). Drawing theoretical inspiration from LC4MP, we propose that structural pacing triggers subjective evaluations of rhythm.

To distinguish this construct from mere perceived complexity, arousal, or general processing load (which lack a sequential temporal dimension), this study defines perceived film and television narrative rhythm as: the audience’s comprehensive judgment on the structural regularity, event density, audiovisual tension, rhythm fluctuations, and difficulty of following the plot of a specific film or television work.

#### 2.1.2 Attention allocation (AA)

Attention allocation is the process by which individuals allocate limited cognitive resources to specific information or tasks. In film and television viewing, attention may be focused on character dialogs, plot clues, visual changes, and sound information, or it may be disturbed by the external environment and other media activities (Zhang et al., 2022; Bezdek and Gerrig, 2017).

Busselle and Bilandzic (2009) distinguish among attentional focus, narrative understanding, emotional engagement, and presence experience, illustrating that attention is an important aspect of narrative engagement but is not equivalent to a complete narrative experience.

This study defines attention allocation as: the concentration of the audience’s attention on the screen content when viewing a film or television work, and the temporary neglect of the external environment, real-life affairs, and other media activities.

#### 2.1.3 Narrative transportation (NT)

Narrative transportation is the state of psychological involvement formed when individuals are exposed to a story. Green and Brock (2000) believe that narrative transportation involves attentional involvement, mental imagery, and experience of the story world. Audiences may form mental representations of characters, scenes, and events during the viewing process and temporarily reduce their attention to the real environment (Appel, 2022).

This study defines narrative transportation as: the subjective experience in which the audience forms vivid mental imagery of the story world and generates a sense of entering the story space, approaching the event scene, or temporarily ignoring the real environment.

#### 2.1.4 Audience emotional immersion (EI)

Audience emotional immersion is the emotional involvement formed by the audience with the characters’ emotions, story events, and overall atmosphere when viewing a film or television work, manifested as emotional resonance, personal emotional resonance, emotional or physiological responses, and emotional continuation after viewing ends (Zhou et al., 2026).

Oliver and Bartsch (2010) point out that entertainment experiences, in addition to immediate pleasure, may also involve being moved, appreciation, and emotional lingering. Jennett et al.’s (2008) study on immersion experience involves attentional involvement, changes in the sense of time, and weakened perception of the real environment.

This study defines audience emotional immersion as: the emotional resonance, emotional or physiological responses, personal emotional resonance, and post-viewing emotional continuation generated by the audience when viewing a specific film or television work.

### 2.2 Research hypotheses

#### 2.2.1 Perceived film and television narrative rhythm and audience emotional immersion

Objective formal features, such as editing pace and event density, act as dynamic structural cues that intertwine with audiences’ information processing routines (Lang, 2000). Taking this a step further, our holistic construct of perceived rhythm incorporates these sub-element cues. From the perspective of the Limited Capacity Model of Motivated Mediated Message Processing (LC4MP), high-density audiovisual stimuli and continuous structural shifts are fundamentally linked to audience orienting responses, which frequently correspond to heightened tension, anticipation, and emotional arousal (Fisher et al., 2018). Although theoretical models suggest that extremely high cognitive load might correlate with a decline in experiential quality, within the conventional boundaries of mainstream film and television viewing, sustained narrative tension generally presents a robust positive association with audiences’ affective involvement. Therefore, this study aims to test the direct statistical association between these components.

*H1*: Perceived film and television narrative rhythm is significantly and positively associated with audience emotional immersion.

#### 2.2.2 The mediating statistical path of attention allocation

Previous psychological research indicates that specific narrative structures, such as suspense, can intensely narrow attentional focus (Bezdek and Gerrig, 2017). Building upon this evidence, we deduce that when audiences perceive a fast-advancing plot, dense conflicts, and distinct rhythm changes, their cognitive processing mechanism typically aligns with a correspondingly higher level of attentional resource allocation. An intense narrative rhythm is usually accompanied by a reduced tendency for external distraction, maintaining a strong statistical connection with sustained continuous screen focus. Simultaneously, this concentration of attentional focus serves as an essential cognitive prerequisite for audiences to successfully interpret character behaviors, dialogs, and subtle audiovisual emotional cues. Consequently, attention allocation is expected to function as an informational bridge between the perception of pacing and emotional engagement.

*H2*: Attention allocation significantly mediates the relationship between perceived film and television narrative rhythm and audience emotional immersion.

#### 2.2.3 The mediating statistical path of narrative transportation

The tight organization of continuous plot clues, conflicts, and soothing segments, alongside coordinated audiovisual tension, provides the necessary informational conditions for audiences to actively construct mental situation models. According to narrative transportation theory (Green and Brock, 2000), immersive situational imagery and the sense of “being there” are closely correlated with uninterrupted narrative inputs. While prior experimental studies demonstrate that variations in sensory modalities and emotional arousal significantly alter time perception during film viewing (Appelqvist-Dalton et al., 2022), we theorize that a cohesive perception of higher narrative rhythm similarly minimizes psychological gaps in the story. This coherence presents a positive link with psychological entry into the narrative space. Furthermore, once a strong sense of presence is formed, this state of vivid mental imagery frequently covaries with intense emotional responses toward the characters.

*H3*: Narrative transportation significantly mediates the relationship between perceived film and television narrative rhythm and audience emotional immersion.

#### 2.2.4 Chain mediation path

Attention allocation and narrative transportation capture interrelated but distinct dimensions of viewer involvement: the former reflects a foundational concentration of cognitive resources driven simultaneously by bottom-up stimulus features and top-down viewing goals (Fisher et al., 2018), while the latter represents a more holistic top-down psychological simulation and spatial immersion (Busselle and Bilandzic, 2009). A sequential structural association may exist between these two states. Maintaining baseline attention on the screen content is statistically tied to the continuous reception of story information, which constitutes the precursor for generating complex mental imagery. Subsequently, this deep psychological entry into the constructed story world intimately correlates with the ultimate experiences of character empathy and overall story atmosphere. Therefore, this study proposes a continuous sequence spanning from cognitive resource allocation to psychological representation, and ultimately to affective experience.

*H4*: Attention allocation and narrative transportation sequentially mediate the relationship between perceived film and television narrative rhythm and audience emotional immersion.

#### 2.2.5 The moderating role hypothesis of trait empathy

Trait empathy (TE) is the tendency of an individual to understand and experience the emotional states of others. In film-media-based viewing experiences, narrative transportation captures the audience’s psychological entry into the story space, while emotional immersion reflects the emotional responses accompanying this psychological entry. According to individual difference processing theory, an individual’s innate emotional sensitivity may present differentiated conditional characteristics in this association. Audiences with high trait empathy, under the same degree of narrative transportation (e.g., equally feeling like they are on the scene), may report higher levels of emotional resonance due to a stronger tendency for fictional involvement and empathic concern (Davis, 1983); conversely, for audiences with low trait empathy, although they pay attention to and understand the story situation, their statistical connection with emotional experience may be relatively weak. Accordingly, this study proposes a moderation hypothesis of trait empathy on the latter half of the chain mediation:

*H5*: Trait empathy positively moderates the relationship between narrative transportation and audience emotional immersion. Specifically, the higher the level of trait empathy, the closer the positive statistical connection between narrative transportation and emotional immersion.

Synthesizing the above literature review and theoretical deductions, this study constructs a theoretical framework encompassing perceived film and television narrative rhythm, attention allocation, narrative transportation, audience emotional immersion, and trait empathy. The related hypotheses regarding direct effects, mediating paths (including independent mediation and chain mediation), and moderating effects are summarized in Figure 1.

**Figure 1:**
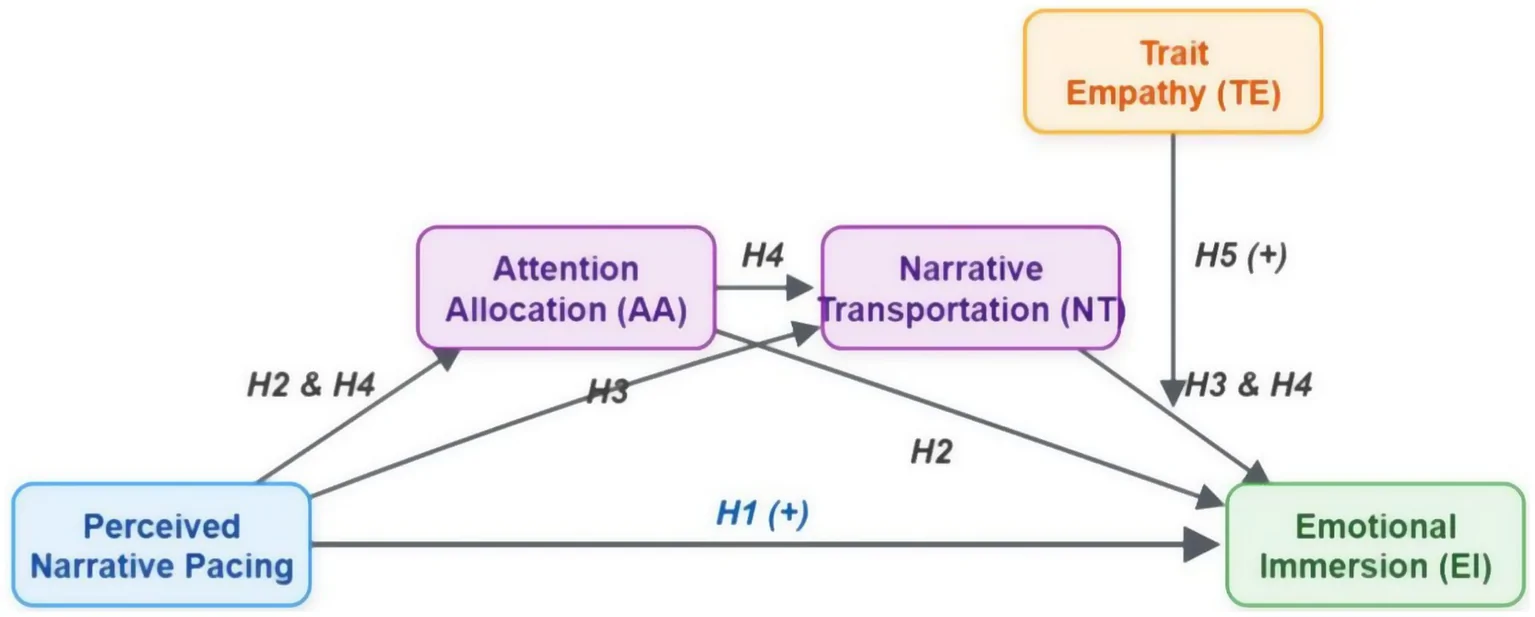
Theoretical framework and hypothesized path diagram of this study.

## 3 Research design and methods

### 3.1 Research sample and data collection

This study utilized cross-sectional survey data, and the research subjects were viewers of film and television content aged 18 and older. The questionnaire asked respondents to recall a film, TV drama, web series, or micro-drama they had recently watched or that left a deep impression on them, and to complete subsequent items based on their viewing experience of that work. The study did not restrict specific platforms, genres of works, or viewing devices.

The questionnaire content included informed consent and age screening, trait empathy, information about the viewed object, core variable measurement, and demographic information. Core variables included perceived film and television narrative rhythm, attention allocation, narrative transportation, and audience emotional immersion; demographic variables included gender, age, education level, main viewing device, and average daily viewing time.

The questionnaire was collected anonymously via a professional online survey platform (Wenjuanxing), and participants received a standard nominal monetary compensation upon successful completion. Respondents participated in the survey on an informed and voluntary basis, with data strictly used for academic research. To maintain consistency in responses, respondents completed the core scales revolving around the same film or television work.

This study collected a total of 1,120 original questionnaires. Data cleaning was conducted based on the responses to informed consent, age screening, and attention check questions, finally retaining 1,074 valid samples, with an effective response rate of 95.89%. The specific distribution of demographic and behavioral characteristics is detailed in Table 1.

**Table 1:** Distribution of demographic and behavioral characteristics.

Variable	Category	Frequency (*n* = 1,074)	Percentage (%)
Gender	Male	458	42.64%
	Female	616	57.36%
Age	18–25 years old	404	37.62%
	26–35 years old	430	40.04%
	36–45 years old	153	14.25%
	46 years old and above	87	8.10%
Education	High school and below	123	11.45%
	Associate degree/Vocational	292	27.19%
	Bachelor’s degree	561	52.23%
	Master’s degree and above	98	9.12%
Main Viewing Device	Smartphone	597	55.59%
	Tablet	185	17.23%
	PC/Laptop	135	12.57%
	Smart TV/Projector	157	14.62%
Avg. Daily Viewing Time	Less than 1 h	162	15.08%
	1–2 h	447	41.62%
	2–4 h	344	32.03%
	More than 4 h	121	11.27%

### 3.2 Measurement tools and scale design

This study utilized a self-report questionnaire to measure respondents’ viewing experiences. All core variables used a 7-point Likert scale, with a scoring range of 1 to 7, where 1 means “completely disagree” and 7 means “completely agree.” A higher score indicates a higher degree of agreement with the corresponding description. The questionnaire items referenced relevant theories and existing studies and were adjusted based on the film and television viewing context. To ensure transparency and reproducibility of the research, the complete list of survey questionnaire items used in this study (including welcome messages, informed consent forms, and instructions for each module) and the detailed Data Codebook have been provided in the Supplementary material.

#### 3.2.1 Trait empathy

The measurement of trait empathy was adapted from the Interpersonal Reactivity Index by Davis (1983), setting a total of 3 items to measure empathic concern, emotional contagion, and tendency to identify with fictional characters. The mean of the three items constitutes the trait empathy variable, denoted as TE_M.

#### 3.2.2 Perceived film and television narrative rhythm

Perceived film and television narrative rhythm referenced the film form study by Bordwell et al. (2008) and the Limited Capacity Model of Mediated Message Processing by Lang (2000), consisting of 5 items assessing plot advancement speed, event and conflict density, audiovisual tension, rhythm fluctuations, and difficulty of following the plot. The mean of the five items constitutes PNR_M.

#### 3.2.3 Attention allocation

The measurement of attention allocation was adapted from the attentional focus subscale developed by Busselle and Bilandzic (2009). To appropriately capture viewing behaviors across multi-dimensional device scenarios, the original items were specifically modified for the current mobile film and television viewing context, consisting of 5 items assessing screen attention, neglect of the external environment, changes in time perception, and the degree of distraction during viewing. Among them, AA4 is a reverse-scored item, and after score conversion, it aligns in the same direction as the other items. The mean of the five items constitutes AA_M.

#### 3.2.4 Narrative transportation

Narrative transportation referenced the narrative transportation theory by Green and Brock (2000), including 4 valid measurement items: NT1, NT2, NT4, and NT5, to measure the sense of entering the story world, situational imagery, sense of witnessing the scene, and neglect of the real environment. NT3 was an attention check question and was not included in the scale score. The mean of the four valid items constitutes NT_M.

#### 3.2.5 Audience emotional immersion

Audience emotional immersion referenced the study on audience emotional responses by Oliver and Bartsch (2010), combined with the discussion on immersion experience by Jennett et al. (2008), setting a total of 4 items to measure character emotional resonance, emotional or physiological responses, personal emotional resonance, and emotional continuation after viewing. The mean of the four items constitutes EI_M.

In addition, the questionnaire included variables such as the name of the work, genre, gender, age, education, main viewing device, and average daily viewing time. The name of the work was an open-ended text variable, while the remaining variables were categorically coded based on the questionnaire options.

### 3.3 Analytical strategy and data preprocessing

This study conducted analyses in the sequence of “data cleaning — descriptive statistics — reliability and validity testing — correlation analysis — regression analysis — indirect and moderation path testing”. Statistical testing adopted two-tailed tests, with the significance level set at 0.05. The specific analysis tools and validation process settings were as follows:

#### 3.3.1 Handling missing values and outliers

After importing the data, variable names, data types, coding ranges, duplicate records, and missing situations were checked. For core items, focus was placed on checking blanks, values outside the 1 to 7 range, and unrecognizable codes.

Cases with many missing core variables or missing responses to informed consent or attention check questions were excluded; when a small number of items were missing, the dimension mean was calculated based on the valid items. Outliers were identified combining *Z*-scores and box plots. Cases with an absolute *Z*-score greater than 3 were listed as potential extreme values, and judgments were made based on response completeness, answering logic, and attention check results. Outliers were not directly deleted based on a single statistical criterion; they were only excluded when confirmed as invalid responses (e.g., intentionally providing contradictory answers to embedded attention check items, displaying uniform straight-lining across all scale items, or submitting the survey in an impossibly short duration).

#### 3.3.2 Variable centering and reverse item scoring

To reduce the potential interference of multicollinearity on the regression models and interaction term tests, this study performed mean centering on the corresponding means of core continuous variables (such as trait empathy, perceived narrative rhythm, narrative transportation, etc.) before conducting non-linear regression and moderator variable analysis. Additionally, AA4 in the attention allocation scale is a reverse item. A higher original score indicated a higher susceptibility to distraction during viewing, which was converted using the following equation:

AA4R = 8 – AA4

Specifically converted as:

1 → 7, 2 → 6, 3 → 5, 4 → 4, 5 → 3, 6 → 2, 7 → 1

Reliability analysis, factor analysis, and dimension mean calculation all used the reversed AA4_R. NT3 was used only for attention screening and was not included in NT_M.

#### 3.3.3 Statistical analysis process

Descriptive statistics included frequencies, percentages, means, and standard deviations, and normality tests were conducted on the core variables. Scale reliability was evaluated using Cronbach’s *α* coefficient; validity testing included exploratory factor analysis and confirmatory factor analysis, focusing on evaluating factor loadings, composite reliability, average variance extracted (AVE), and discriminant validity. Meanwhile, to address common method bias (CMB) common in questionnaire methods (Podsakoff et al., 2003), based on using Harman’s single-factor test, a secondary check was performed by constructing a structural model incorporating an Unmeasured Latent Method Construct (ULMC) via AMOS. Descriptive statistics, correlation analysis, and non-linear hierarchical regression, and preliminary difference tests on recalled narrative genres were completed using SPSS; CFA and AMOS were used to test the measurement model and the core structural model, reporting fit indices such as *χ*^2^/*df*, RMSEA, CFI, and TLI. To explicitly account for potential confounding effects, demographic variables and trait empathy were parameterized as exogenous covariates within the main structural equation model.

For the testing of indirect associations and moderation paths, the mediation analysis based on the PROCESS macro program (Hayes, 2013) set perceived film and television narrative rhythm as the explanatory variable, attention allocation and narrative transportation as mediator variables, audience emotional immersion as the outcome variable, and controlled for trait empathy, gender, age, education, main viewing device, and average daily viewing time. Bootstrap resampling was performed 5,000 times, using the 95% confidence interval to determine indirect associations.

## 4 Data analysis and hypothesis testing

### 4.1 Data quality and sample characteristics

This study finally retained 1,074 valid questionnaires. Harman’s single-factor test was conducted on the 21 core measurement items; the explanatory variance of the first principal component was 32.13%, which is lower than the reference standard of 40%, indicating no obvious common method bias. For further rigorous diagnosis, this study compared the fit indices of the ULMC model after controlling for a global method latent variable (CFI = 0.996, RMSEA = 0.015) with the original base measurement model (CFI = 0.994, RMSEA = 0.017). The changes in key fit indices were extremely minimal (ΔCFI = 0.002, ΔRMSEA = 0.002), far below the widely accepted reference threshold of 0.05. Synthesizing the above evidence indicates that the data obtained in this study does not suffer from severe common method bias.

The five core variables were all calculated using item means. The means of each variable ranged from 4.733 to 4.801, and standard deviations ranged from 1.029 to 1.106. The absolute values of skewness and kurtosis for all variables were less than 1, indicating no significant skew. K-S tests all reached statistical significance. Considering that the K-S test is highly sensitive to slight deviations under large sample conditions, and combining skewness, kurtosis, and sample size, Pearson correlation, regression analysis, and structural equation modeling were subsequently adopted. The descriptive statistics and normality test results for each core variable are shown in Table 2.

**Table 2:** Descriptive statistics and normality tests of core variables.

Variable	*N*	*M*	*SD*	Skewness	Kurtosis	K-S D	*p*
Trait Empathy (TE)	1,074	4.801	1.048	−0.183	−0.252	0.0733	<0.001
Perceived Narrative Rhythm (PNR)	1,074	4.733	1.029	−0.211	−0.164	0.0530	0.005
Attention Allocation (AA)	1,074	4.760	1.036	−0.173	−0.399	0.0563	0.002
Narrative Transportation (NT)	1,074	4.786	1.106	−0.191	−0.295	0.0533	0.004
Emotional Immersion (EI)	1,074	4.738	1.063	−0.271	−0.053	0.0558	0.002

Demographic difference analysis used audience emotional immersion as the dependent variable. An independent samples t-test was used for gender, and one-way ANOVA was used for age, education, viewing device, daily viewing time, and recalled narrative genre.

The results showed that the difference between gender groups was not significant, *t* = −0.093, *p* = 0.926, *η*^2^ < 0.001. The between-group differences for age, education, viewing device, daily viewing time, as well as narrative genre, all failed to reach statistical significance, and the effect sizes for all variables were less than 0.01. Despite the absence of significant main effects, to rigorously eliminate any potential systematic baseline variations, these demographic characteristics and trait empathy were strictly retained as exogenous control covariates in all subsequent structural models. The specific results of demographic between-group difference tests are detailed in Table 3.

**Table 3:** Demographic variable difference tests.

Variable	Test method	Statistic	*p*	*η* ^2^
Gender	Independent samples *t*-test	−0.093	0.926	<0.001
Age	One-way ANOVA	1.654	0.175	0.005
Education	One-way ANOVA	1.086	0.354	0.003
Viewing device	One-way ANOVA	0.792	0.498	0.002
Daily viewing time	One-way ANOVA	1.467	0.222	0.004

### 4.2 Measurement model testing

The study items referenced relevant theories and existing scales, and were adjusted combined with the film and television viewing context. Because the current data only contains respondents’ completion results and does not include expert scoring data, the Content Validity Index (CVI) was not numerically calculated.

An exploratory factor analysis was conducted on the 21 core items. The KMO value was 0.914, and the Bartlett’s test of sphericity showed significant results, *χ*^2^ = 8561.72, *df* = 210, *p* < 0.001, indicating the data were suitable for factor analysis. The analysis extracted 5 factors, which aligned with the theoretical structures of trait empathy, perceived narrative rhythm, attention allocation, narrative transportation, and audience emotional immersion. The maximum absolute factor loadings of the items ranged from 0.62 to 0.80. Specific indicators and results of the exploratory factor analysis are shown in Table 4.

**Table 4:** Exploratory factor analysis results.

Indicator	Result
KMO	0.914
Bartlett’s Test of Sphericity *χ*^2^	8561.720
*df*	210
*p*	<0.001
Number of Factors Extracted	5
Maximum Absolute Factor Loading	0.620–0.804

The results of the confirmatory factor analysis showed that for the five-factor model: *χ*^2^ = 232.491, *df* = 179, *χ*^2^/*df* = 1.299, RMSEA was 0.017, CFI was 0.994, and TLI was 0.993 and SRMR was 0.021; all model fit results met common reference standards (Hu and Bentler, 1999).

The Cronbach’s *α* coefficients of the scales ranged from 0.738 to 0.836, and the composite reliability (CR) ranged from 0.739 to 0.836. The Average Variance Extracted (AVE) for trait empathy, perceived narrative rhythm, and attention allocation were 0.487, 0.491, and 0.494 respectively; the AVE for narrative transportation and emotional immersion were 0.560 and 0.538, respectively. The AVE values for the first three constructs were slightly below 0.50 but close to the standard. Combining the results of factor loadings, CR, and discriminant validity, the measurement quality of the scales is within an acceptable range. The reliability, convergent validity, and AVE indicators of each scale are summarized in Table 5.

**Table 5:** Reliability, convergent validity, and discriminant validity.

Construct	Cronbach’s *α*	CR	AVE	Square root of AVE
Trait Empathy (TE)	0.738	0.739	0.487	0.698
Perceived Narrative Rhythm (PNR)	0.827	0.828	0.491	0.701
Attention Allocation (AA)	0.829	0.829	0.494	0.703
Narrative Transportation (NT)	0.836	0.836	0.560	0.749
Emotional Immersion (EI)	0.822	0.823	0.538	0.733

The square roots of AVE for all constructs were higher than the correlation coefficients between that construct and other constructs. The maximum correlation coefficient between constructs was 0.526, lower than the square root of the AVE for each corresponding construct. According to the Fornell-Larcker criterion (Fornell and Larcker, 1981), discriminant validity was supported.

### 4.3 Core variable relationship analysis

Pearson correlation analysis showed significant positive correlations among the five core variables. The correlation coefficients between perceived narrative rhythm and attention allocation, narrative transportation, and emotional immersion were 0.418, 0.406, and 0.354, respectively; the correlation coefficients between attention allocation and narrative transportation, and emotional immersion were 0.451 and 0.426, respectively; the correlation coefficient between narrative transportation and emotional immersion was 0.526. All correlation coefficients reached the *p* < 0.001 level.

The correlation coefficients between trait empathy and attention allocation, narrative transportation, and emotional immersion were 0.256, 0.275, and 0.302, respectively, so it was included as a covariate in subsequent models. The Pearson correlation matrix among the core variables is detailed in Table 6.

**Table 6:** Pearson correlation matrix of core variables.

Variable	TE	PNR	AA	NT	EI
TE	1.000	0.208	0.256	0.275	0.302
PNR	0.208	1.000	0.418	0.406	0.354
AA	0.256	0.418	1.000	0.451	0.426
NT	0.275	0.406	0.451	1.000	0.526
EI	0.302	0.354	0.426	0.526	1.000

All correlation coefficients reached the *p* < 0.001 level.

A hierarchical regression was conducted with audience emotional immersion as the dependent variable. Model 1 included demographic variables and trait empathy, *R*^2^ = 0.103, Adjusted *R*^2^ = 0.091. After adding perceived narrative rhythm in Model 2, *R*^2^ = 0.191, Adjusted *R*^2^ = 0.180, and the increment in explanatory power was Δ*R*^2^ = 0.089. The unstandardized regression coefficient for PNR was *B* = 0.316, *p* < 0.001.

After controlling for demographic variables and trait empathy, perceived narrative rhythm showed a significant positive association with emotional immersion, supporting H1.

Furthermore, to test the potential “cognitive overload” threshold issue in the Limited Capacity Model of Motivated Mediated Message Processing (LC4MP), this study introduced the centered perceived narrative rhythm (PNR_c) and its squared term (PNR_sq) into Model 3 to perform a hierarchical non-linear regression. The analysis results showed: the regression coefficient of the squared term was extremely small and completely non-significant (*B* = 0.003, *p* = 0.891), and the systemic variance explained increment was virtually zero (Δ*R*^2^ = 0.000). This result essentially rejected the common theoretical presumption of an “inverted U-shaped association between perceived narrative rhythm and immersion,” indicating that within the respondents’ normal film and television viewing range, perceived narrative rhythm maintains a robust positive linear association with audience emotion. The detailed hierarchical regression and non-linear test results are integrated in Table 7.

**Table 7:** Hierarchical regression analysis results.

Model	Included variables	*R* ^2^	Adjusted *R*^2^	Δ*R*^2^	*B* value of PNR	*p*
Model 1	Demographic variables, Trait empathy	0.103	0.091	–	–	–
Model 2	Model 1 + PNR	0.191	0.180	0.089	0.316	<0.001
Model 3	Model 1 + PNR_c + PNR_sq (Squared term)	0.191	0.179	0.000	0.003 (PNR_sq)	0.891

As shown in the full-sample scatter plot and fitting trends in Figure 2, the non-linear curve introducing the quadratic term almost completely overlaps with the basic linear regression trajectory, intuitively confirming that within the current observation range, perceived narrative rhythm consistently maintains a robust positive linear association with audience emotional immersion, and the “inverted U-shaped” experience decay inflection point caused by cognitive overload was not observed.

**Figure 2:**
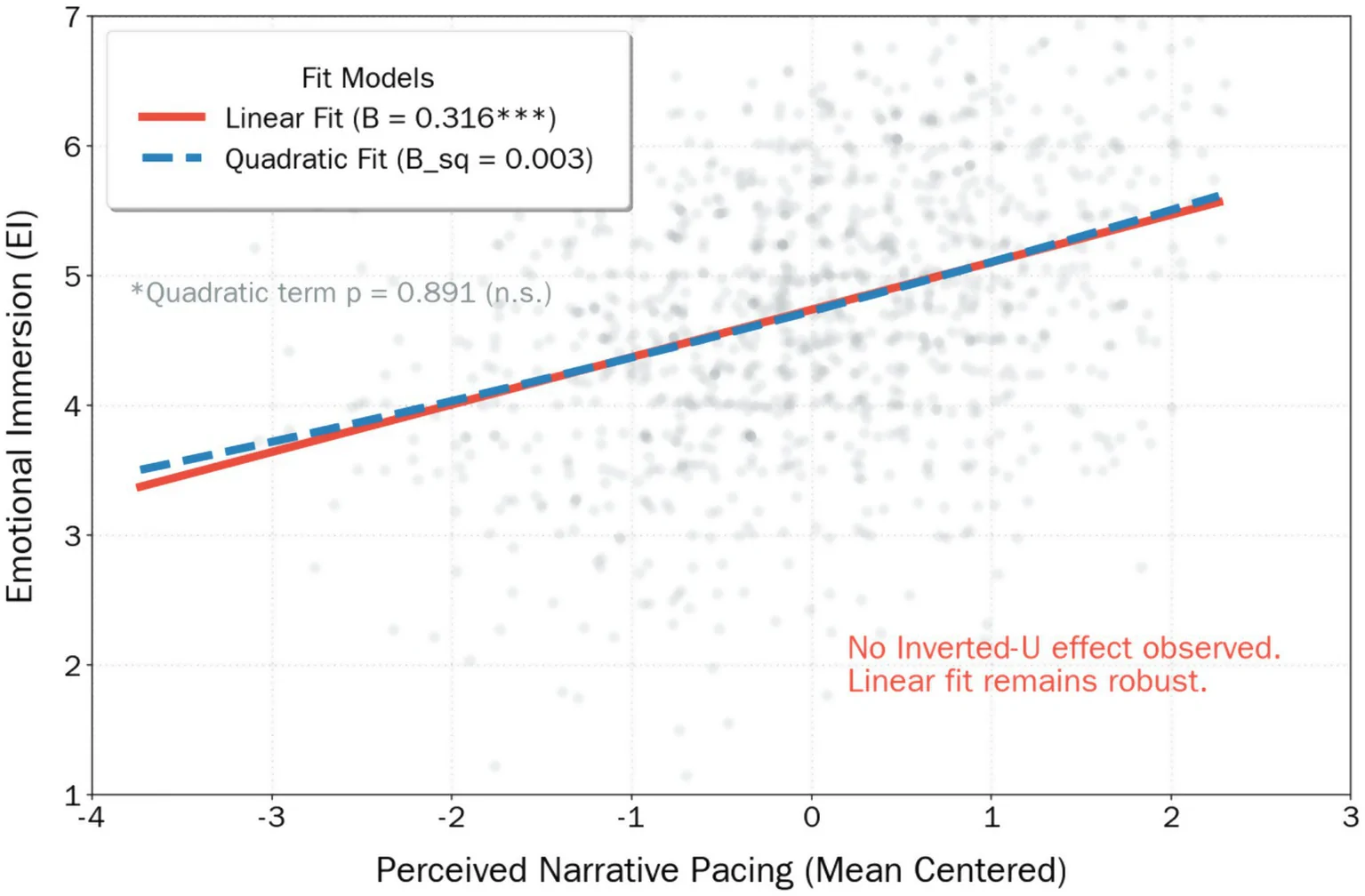
Comparison of linear and non-linear (inverted U-shaped) fitting trends of perceived narrative rhythm and audience emotional immersion.

### 4.4 Mediation path and hypothesis testing

The mediation model set perceived narrative rhythm as the explanatory variable, attention allocation and narrative transportation as mediator variables, and audience emotional immersion as the outcome variable, controlling for trait empathy, gender, age, education, main viewing device, and daily viewing time. Bootstrap resampling was performed 5,000 times, using whether the 95% confidence interval contained 0 to judge the indirect association.

The estimated indirect association via attention allocation was 0.0778, 95% CI [0.0532, 0.1055]; the estimated association via narrative transportation was 0.0929, 95% CI [0.0682, 0.1203]; the estimated chain association via attention allocation and narrative transportation was 0.0453, 95% CI [0.0335, 0.0590]. The confidence intervals of all three indirect paths did not contain 0, supporting H2, H3, and H4. The specific Bootstrap indirect association test estimation results are detailed in Table 8.

**Table 8:** Bootstrap indirect association test.

Path	Association estimate	Boot SE	95% CI lower limit	95% CI upper limit	Result
PNR → AA → EI	0.0778	0.0133	0.0532	0.1055	Supported H2
PNR → NT → EI	0.0929	0.0132	0.0682	0.1203	Supported H3
PNR → AA → NT → EI	0.0453	0.0067	0.0335	0.0590	Supported H4

The structural equation model (which strictly incorporated demographic variables and trait empathy as exogenous control covariates) demonstrated a robust overall fit to the data (*χ*^2^/*df* = 1.05, RMSEA = 0.007, CFI = 0.998, TLI = 0.998, SRMR = 0.015). The estimation results showed that the standardized path coefficient between PNR and AA was 0.462, between PNR and NT was 0.259, and between AA and NT was 0.358, all reaching the *p* < 0.001 level. In the EI equation, the standardized path coefficient for AA was 0.203, and for NT was 0.436, both reaching the *p* < 0.001 level.

After incorporating AA and NT, the residual direct path between PNR and EI was 0.071, *p* = 0.069. This path constitutes the residual direct association after simultaneously incorporating the mediator variables, corresponding to a different model specification than the overall association of PNR on EI in the hierarchical regression. As comprehensively illustrated in the structural equation model path diagram (Figure 3), after fully incorporating attention allocation and narrative transportation, the residual direct association between perceived narrative rhythm and audience emotional immersion is no longer significant (*β* = 0.071, n.s.), and a significant chain indirect statistical association structure presents between the two.

**Figure 3:**
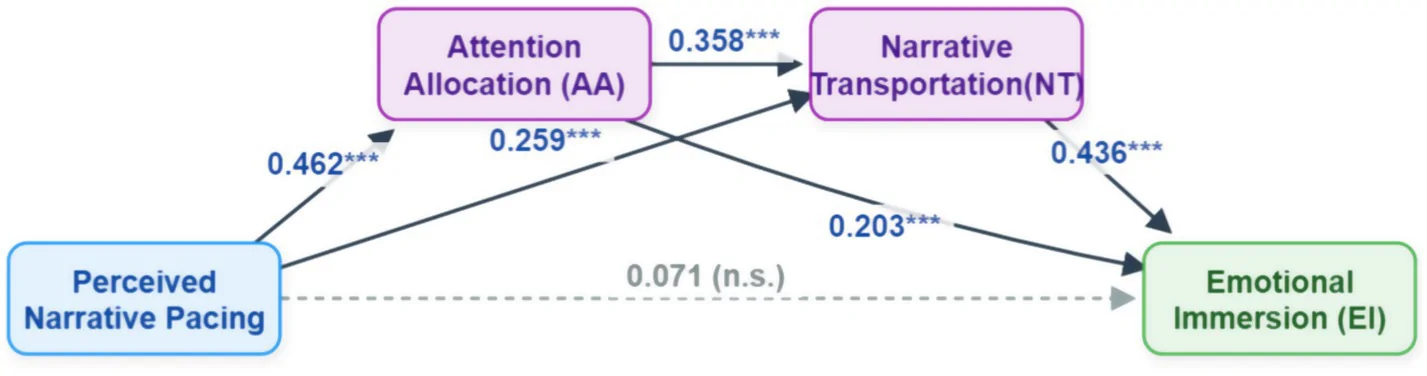
Structural equation model (SEM) empirical path estimation results for audience emotional immersion. Values are standardized path coefficients. ***p < 0.001.

### 4.5 Moderation hypothesis testing of trait empathy

To test the moderating role of trait empathy in the core association structure (H5), this study used a multiple partial regression model to conduct an interaction term analysis. To eliminate the impact of multicollinearity, the continuous independent variables in the model were all pre-processed with mean centering.

The regression coefficient of trait empathy (TE) on emotional immersion was significant (*B* = 0.136, *SE* = 0.026, *p* < 0.001), indicating that audiences with high trait empathy reported a higher overall emotional involvement baseline. However, the predictive effect of the interaction term between narrative transportation and trait empathy (NT_x_TE) on emotional experience did not reach statistical significance (*B* = −0.003, *SE* = 0.022, *p* = 0.888). This indicates that trait empathy did not significantly moderate the association between narrative transportation and emotional immersion; that is, the statistical association strength on this path did not differ significantly for audiences of different trait empathy levels. Synthesizing the above analysis, Hypothesis H5 was not supported by the sample data. The specific coefficients and significance of the interaction term analysis are shown in Table 9.

**Table 9:** Interaction term analysis results.

Predictor variable	Unstandardized coefficient *B*	Standard error SE	*t*-value	*p*-value	[95% confidence interval]
Constant	3.062	0.235	13.026	<0.001	[2.601, 3.523]
Gender	0.051	0.053	0.952	0.341	[−0.054, 0.155]
Age	0.072	0.029	2.506	0.012	[0.016, 0.128]
Education	0.029	0.032	0.898	0.369	[−0.035, 0.093]
Viewing device	0.020	0.024	0.864	0.388	[−0.026, 0.067]
Daily time	−0.022	0.030	−0.716	0.474	[−0.081, 0.038]
PNR (main effect control)	0.102	0.029	3.492	0.001	[0.045, 0.159]
AA (main effect control)	0.192	0.030	6.418	<0.001	[0.133, 0.251]
NT (narrative transportation)	0.355	0.028	12.620	<0.001	[0.300, 0.410]
TE (trait empathy)	0.136	0.026	5.161	<0.001	[0.085, 0.188]
NT × TE (interaction term)	−0.003	0.022	−0.141	0.888	[−0.047, 0.041]

All core continuous variables (NT, TE) and their interaction terms were included in the model after mean centering.

As intuitively demonstrated by the parallel slope graph in Figure 4, although the overall emotional baseline of audiences with high trait empathy (+1 SD) was significantly higher than that of audiences with low trait empathy (−1 SD), the “narrative transportation—emotional immersion” regression slope lines under the two different trait levels remained highly parallel and did not intersect, visually confirming that this segment of the mechanism path possesses homogeneous stability that is not significantly modified by trait sensitivity.

**Figure 4:**
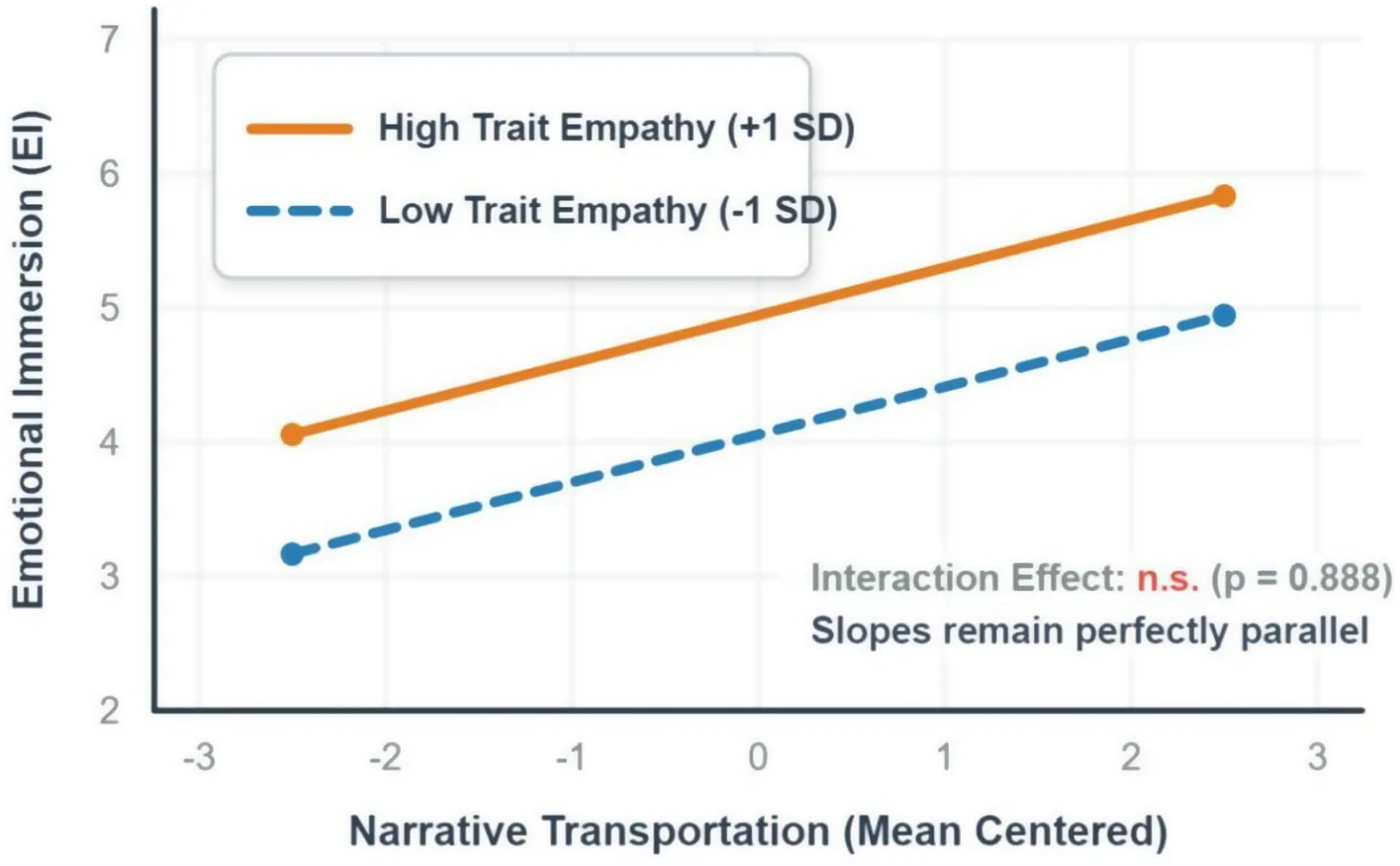
Parallel slope graph of the association between narrative transportation and emotional immersion across high/low trait empathy levels.

## 5 Discussion

### 5.1 Association between perceived narrative rhythm and emotional immersion

This study found that audience evaluation of film and television narrative rhythm is positively associated with emotional immersion. Given the multifaceted nature of rhythm perception, this finding conceptually implies that the audience’s judgment extends beyond a simple evaluation of visual fastness or slowness. Rather, it likely acts as a comprehensive proxy reflecting their overall perception of information organization, plot continuity, and audiovisual changes.

It should be pointed out that based on the Limited Capacity Model of Motivated Mediated Message Processing (LC4MP; Lang, 2000; Fisher et al., 2018), some studies presupposed that when information density and rhythm exceed the audience’s cognitive load, emotional experience might correspondingly decline (i.e., presenting an inverted U-shaped relationship). However, the quadratic regression results of this study showed that the coefficient of the squared term for perceived narrative rhythm was extremely small and insignificant, and the two maintained a stable linear positive association within the sample of this study. This may imply that within the normal film and television rhythm range encountered by respondents, the more compact textual information has not yet exceeded their comprehension burden threshold. Audiences have a certain adaptation space for coherent and high-information-density narratives, which provides the possibility for the coexistence of higher rhythm scores and positive emotional involvement indicators.

### 5.2 The mediating statistical path of attention allocation

Attention allocation presents an indirect statistical path between perceived narrative rhythm and emotional immersion, indicating that there is a connection between audiences’ perception of rhythm and their attention resource allocation.

Film and television viewing requires audiences to continuously process character dialogs, plot clues, visual changes, and sound information (Zhang et al., 2022). For works where information advancement is relatively concentrated and conflict relations are relatively clear, audiences may find it easier to maintain attention on the screen content. Concentration of attention can help audiences continuously receive narrative information and provide the necessary information base for subsequent emotional experiences.

However, attention allocation is not equivalent to emotional immersion. Audiences might maintain high attention to understand complex plots but not form obvious emotional resonance; or they may report high-level emotional reactions due to character relationships, life experiences, or specific plots even when attention is not continuously concentrated (Oliver and Bartsch, 2010). Therefore, attention allocation is closer to cognitive involvement during the viewing process, while emotional immersion reflects the experience outcome at the emotional level.

This study found that the AA→EI path was significant, indicating a statistical association between maintaining attention on film and television content and emotional immersion. This empirical result aligns with fundamental attentional principles and the core premises of the limited capacity information processing perspective (Lang, 2000), confirming that adequate allocation of baseline cognitive resources to media content serves as a crucial informational prerequisite for actively receiving and processing characters, events, and audiovisual cues.

### 5.3 The mediating statistical path of narrative transportation

Narrative transportation presents an indirect statistical path between perceived narrative rhythm and emotional immersion, and the indirect effect of this path is higher than the single path via attention allocation. This indicates that story world experience may be an important link connecting narrative rhythm perception and emotional immersion (Wang and Tang, 2021).

Film and television works provide story information to the audience through character actions, scene settings, dialogs, sounds, and plot relationships. After receiving this information, audiences may form mental representations of the story space, character situations, and event scenes. When audiences generate experiences such as “entering the story,” “approaching the event scene,” or “temporarily ignoring the real environment” (Green and Brock, 2000), character behaviors and plot conflicts may more easily form close statistical connections with specific emotional feeling scores.

The significant association between narrative transportation and emotional immersion indicates that the audience’s emotional experience depends not only on whether they focus on the screen but also on whether a relatively complete story world experience has been formed. This result also demonstrates that the role of film and television narrative rhythm may not be limited to speeding up or slowing down information transmission. Rhythm arrangement may also affect the continuity of story clues, the completeness of situational construction, and the understanding of character relationships, thereby linking with the audience’s narrative transportation level.

### 5.4 The chain path of attention allocation and narrative transportation

This study found that a chain indirect association exists among perceived narrative rhythm, attention allocation, narrative transportation, and emotional immersion. This finding supports the theoretical sequence proposed in this study: audiences’ perception of narrative rhythm is positively associated with attention allocation, which in turn relates to higher narrative transportation, ultimately corresponding to greater emotional immersion.

From the perspective of viewing experience, audiences need to maintain attention to the screen content to a certain extent in order to continuously receive character dialogs, plot clues, and audiovisual changes. Continuously receiving narrative information helps audiences form mental representations of the story space and character situations. Story world experience is further related to emotional resonance, personal emotional connection, and emotional continuation after viewing (Jennett et al., 2008).

The statistical results of the chain path show that attention allocation and narrative transportation hold different positions in the model. Attention allocation is closer to the allocation of cognitive resources, and narrative transportation is closer to psychological involvement in the story world. After fully incorporating both into the model, it can more clearly distinguish the two interrelated but distinct viewing processes of “focusing on the work” and “entering the story” (Busselle and Bilandzic, 2009). It is worth mentioning that after the structural equation model fully incorporates this group of chain nodes, the residual direct path coefficient of perceived narrative rhythm to emotional immersion attenuated significantly to a marginally significant level (*β* = 0.071, *p* = 0.069). This structural distribution, approximating complete mediation, suggests that high-intensity film and television rhythm itself does not blindly equate to the acquisition of deep emotional experience. The effectiveness of rhythmic form lies in whether it can match the audience’s attentional resources and smoothly transition to internal logical construction (i.e., narrative transportation); this provides a quantitative psychological explanation for the common critique of films having “a fast pace but lacking narrative appeal.”

By validating the chain mediation model of “perceived narrative rhythm → attention allocation → narrative transportation → emotional immersion,” this study preliminarily outlines the sequence of psychological processing indicators for audiences viewing high-rhythm film and television works. This path reveals the bottom-up connection characteristics of audiovisual media consumption: first, prominent rhythm features are positively linked with the audience’s high-level screen focus (attention allocation) scores; second, this cognitive attention state further covaries with psychological simulation indicators of the story space (narrative transportation); ultimately, these coherent viewing states collectively build the data foundation for the audience’s high-level emotional feedback (emotional immersion) (Bezdek and Gerrig, 2017). This model not only provides empirical clues for “formal features associated with implicit psychological responses” but also offers a new theoretical explanation for understanding the cognitive processing sequence of audience media information processing in the mobile internet era.

### 5.5 Theoretical implications of the unsupported moderation effect and analysis of control variables

From a theoretical presupposition standpoint, trait empathy holds a significant positive correlation with various indicators of audience immersion experience, making its inclusion necessary. However, this study hypothesized that trait empathy would positively moderate the association strength of narrative transportation translating into emotional immersion (H5), and this expectation was not supported by the cross-sectional data.

The moderated mediation effect model showed that the main effect of trait empathy on emotional immersion was significant (indicating that audiences with higher trait empathy inherently possess a higher emotional response baseline norm), but its interaction term with narrative transportation was extremely weak and failed to significantly predict emotional immersion (*β* = −0.003, *p* = 0.888, H5 unsupported). This non-significant result offers specific theoretical enlightenment: although there are high and low differences in individuals’ innate empathy levels—which constitutes the natural baseline of their emotional responses—the statistical association structure of “narrative transportation → emotional immersion” maintained a high degree of parallelism and stability across different trait populations. This indicates that once film and television narrative rhythm is accompanied by audience attention and involvement in the story space, the statistical path strength extending toward emotional feedback demonstrates strong universality and internal consistency, and is no longer subject to significant modification fluctuations by individual trait sensitivity. However, the robust simple correlation observed between trait empathy and narrative transportation (*r* = 0.275) highlights an alternative theoretical possibility: innate empathy levels might directly elevate the baseline intensity of narrative transportation itself, rather than moderating its subsequent translation into emotional experience. Additionally, while the recalled narrative genres in this sample did not demonstrate significant main effects on emotional immersion, individual trait empathy might still subtly dictate the types of emotional narratives viewers naturally gravitate toward, representing a complex interplay worthy of future exploration.

In terms of demographic indicator controls, difference analysis showed that the between-group main effects for gender, age, education, main viewing device, and daily viewing time on overall emotional immersion all failed to reach statistical significance. This result suggests that in the sample of this study, the deep psychological involvement accompanied by high-intensity perceived rhythm does not center on simple demographic groupings (e.g., gender opposition or viewing terminal differences). Of course, the fact that these peripheral variables do not reflect main differences does not mean they are irrelevant in all segmented film and television consumption contexts. Because this study had properly statistically controlled for them as covariates in the core mechanism testing, the above core chain and mainline paths still demonstrated cross-group structural stability after removing these potential confounding factors.

### 5.6 Theoretical significance

The theoretical significance of this study is reflected in four aspects:

First, the study expanded film and television narrative rhythm from objective formal features to the level of audience subjective perception. Shot length, event quantity, and editing speed can describe the formal structure of a work (Bordwell et al., 2008), but cannot fully explain how audiences experience rhythm. Perceived film and television narrative rhythm can reflect audiences’ comprehensive judgments of speed, density, tension, and fluctuation, providing a measurement path for bridging film and television text research with audience research.

Second, the study distinguished among three constructs: attention allocation, narrative transportation, and emotional immersion. All three can be summarized as “involvement,” but correspond, respectively, to cognitive attention, story world experience, and emotional response. Incorporating them into the same model helps to clarify their statistical positions among different viewing experiences, reducing the problem of conflating concentration of attention, entering the story, and generating emotional responses.

Third, combining the limited capacity information processing perspective with narrative transportation theory, the study constructed a mediation model between perceived narrative rhythm and emotional immersion. The results show that attention allocation and narrative transportation appear, respectively, and sequentially in the statistical association structure of the two, providing a model foundation for subsequent research to further investigate the relationship between film and television narrative rhythm and viewing experience.

Fourth, this study explored the continuous linear pattern between perceived narrative rhythm and experiential involvement through quadratic term analysis, and did not observe a significant inverted U-shaped non-linear deviation within the testing range; simultaneously, through the moderation effect model, it showed that the statistical path from narrative transportation to emotional experience features consistency across trait empathy levels. These results constitute a theoretical supplement to the discussion of the association between information load and mental processing in mobile viewing contexts (Fisher et al., 2018).

### 5.7 Practical significance

For film and television creators, the research results suggest that narrative rhythm design could benefit from a holistic approach. These findings empirically echo the proposition that plot advancement and information density should ideally be coordinated with character shaping, story continuity, and situational construction, rather than merely piling up raw conflict quantities (Zou et al., 2025). Faster rhythms are associated with rich information presentation, but may also be accompanied by a rise in audience understanding pressure; slower rhythms require maintaining audience story involvement through character relationships, situational atmospheres, and emotional cues. In specific practice, as long as the internal logic of information organization remains coherent, audiovisual rhythm of a certain intensity can also be accepted by audiences and provide conditions for subsequent emotional immersion.

For streaming platforms and mobile content producers, the framework design of audiovisual content, audience attention allocation, and narrative transportation jointly construct critical dimensions for evaluating viewing experiences (Appel and Mengelkamp, 2022; Gan et al., 2023; Wang et al., 2023). The arrangement of opening information, segment duration, suspense settings, and configuration of plot nodes can be adjusted combining the audience’s subjective feedback on rhythm. Platform data such as view counts, completion rates, and interaction volume can reflect viewing behavior, but cannot completely replace audiences’ subjective reports on attention, story experience, and emotional responses.

For film and television content evaluation, perceived narrative rhythm can serve as a supplementary variable beyond textual and behavioral indicators. Content testing should not only ask whether the work has a “fast rhythm,” but can also separately examine plot advancement, information density, audiovisual tension, rhythm fluctuations, attention maintenance, and story world experience. To further translate these subjective insights into precise operational blueprints for industry creators, future experimental studies could systematically manipulate physical pacing metrics against narrative information density, thereby establishing clearer causal design strategies.

## 6 Research limitations and future directions

### 6.1 Research limitations

This study adopted a cross-sectional survey, with all variables measured at the same time point, unable to determine the chronological order among perceived narrative rhythm, attention allocation, narrative transportation, and emotional immersion. Mediation analysis results reflect indirect statistical associations and should not be interpreted as confirmed causal relationships. Reverse models and alternative models still need to be verified. For instance, while the current theoretical framework posits that narrative transportation predicts emotional immersion, it is equally plausible that psychological entry into the story world is, in part, a cognitive byproduct of intense emotional engagement. Such alternative directionality requires rigorous testing through longitudinal or cross-lagged panel designs.

The study used a work-recall method, and respondents’ answers may have been subject to memory bias, recency bias, and selection preferences. The works recalled by different respondents varied in genre, duration, platform, viewing environment, and viewing time, and these factors might have covaried as confounding variables with the audience’s rhythm and emotional evaluations. Particularly, the current structural equation model did not systematically incorporate explicit features of the narrative—such as the distinctive viewing formats (e.g., short-form versus long-form content)—as modeling covariates. Because the independent influence of these media features was not empirically estimated, the precision of identifying content-specific rhythmic effects remains somewhat limited.

Core variables were measured using self-report scales, and data might have been subject to social desirability, common method bias, and response styles. Although this study introduced an Unmeasured Latent Method Construct (ULMC) model to attempt assessing the impact of Common Method Bias (CMB) based on Harman’s single-factor test, showing that systematic bias was within an acceptable range, the inherent methodological limitations regarding common method bias in self-report surveys have not been completely eliminated.

Trait empathy was measured using only three items, making it difficult to cover complete dimensions such as perspective-taking, empathic concern, personal distress, and fantasy. The study controlled for some demographic variables and viewing behavior variables, but factors like genre preference, past viewing experience, emotional state during viewing, work quality evaluation, and viewing environment could still impact the relationships among variables.

The AVE values for some constructs in this study were slightly below 0.50, indicating that the measurement tools still have room for further revision. The Content Validity Index could not be calculated based on the current data; subsequent research should add expert peer-review and pre-testing procedures before formal surveys.

### 6.2 Future directions

Subsequent research could adopt experimental designs, providing respondents with film and television clips that differ in rhythm features, and measuring attention, narrative transportation, and emotional experience during the viewing process to further test the chronological order and conditional relationships among the variables.

Additionally, while the perceived narrative rhythm and emotional immersion in this study’s sample ultimately predominantly presented a linear association (failing to observe an inverted U-shaped load inflection point), this might be constrained by the normal rhythm settings of the daily film and television works recalled by the respondents. Future experimental studies could attempt multiple comparisons using audiovisual stimuli with higher information densities or even discontinuous rhythms to explore the boundaries of audience cognitive limits.

Longitudinal studies could conduct multiple measurements before, during, and after viewing to examine changes in perceived narrative rhythm, attention allocation, narrative transportation, and emotional immersion at different stages. Experience sampling and viewing logs could also be used to reduce memory bias, recording immediate experiences under various viewing contexts.

Subsequent studies could also combine textual analysis indicators such as average shot length, scene transition frequency, dialog speed, event density, and sound variations (Bordwell et al., 2008; Cutting et al., 2010), integrating both objective narrative rhythm and perceived narrative rhythm into the model simultaneously to compare the relationship between a work’s formal features and the audience’s subjective judgments.

In terms of measurement, the trait empathy scale could be further expanded; variables like genre familiarity, work evaluation, character identification, viewing environment, viewing purpose, and multitasking behaviors could be added, and the scale structure improved through expert peer-review, pre-testing, and cross-sample validation.

Different film and television genres, platforms, and viewing devices may correspond to different rhythmic experiences. Subsequent research could carry out multi-group analyses comparing model differences among films, TV dramas, web series, and micro-dramas, and could also examine variable relationships under scenarios of mobile viewing, television viewing, and computer viewing (Appel and Mengelkamp, 2022). Overall, future empirical explorations should further reveal: the audience experience of film and television narrative rhythm is not merely about the “fast or slow” of the text, but more importantly about how audiences enter the story world both cognitively and emotionally.

## Data Availability

The original contributions presented in the study are included in the article/Supplementary material, further inquiries can be directed to the corresponding author.
