# Supplementary material for: Linking perceived film and television narrative rhythm with audience emotional immersion: the mediating paths of attention allocation and narrative transportation
Source: Front Psychol. 2026 Oct 28;17:1971219. doi: 10.3389/fpsyg.2026.1971219 (PMC13751338; doi:10.3389/fpsyg.2026.1971219)
Supplement: Supplementary file 1 [file Data_Sheet_1.ZIP › Supplementary_Material.docx]

**Appendix A: Original Measurement Tools of the Survey Questionnaire**

**Part 1: Welcome message and informed consent**

Dear participant, hello! Thank you very much for taking your valuable time to assist with our research. We are a research team focusing on media psychology and film/television creation. Through this short survey, we hope to listen to your authentic inner experiences when watching films, TV dramas, or micro-dramas in your daily life.

This questionnaire is completed independently and anonymously. All data you provide will be kept strictly confidential and used only for statistical analysis in academic research. It will never be used for any commercial purposes or leak your personal privacy. The survey does not involve any sensitive content or content that causes psychological discomfort. As the questions are entirely about your personal subjective feelings, there are no standard right or wrong answers. Please feel free to answer based on your first instinct.

Completing this questionnaire will take about 3-5 minutes. At any point during the answering process, you have the right to withdraw at any time, which will not cause any adverse effects on you. Thank you again for your kind support!

To strictly abide by academic ethical norms and protect the rights and interests of minors, this survey only invites adult friends aged 18 and above to participate.

**1. Are you 18 years of age or older, and do you voluntarily agree to participate in this questionnaire survey?**

I am 18 years old or older and participate voluntarily

I am under 18 years old or it is currently inconvenient to participate (Selecting this option will terminate the survey)

**Part 2: Baseline measurement of daily psychological traits**

Instructions: First, please rate the following descriptions based on your general preferences and personality traits in daily life.

(1 = *Completely disagree*, 2 = *Moderately disagree*, 3 = *Slightly disagree*, 4 = *Neutral*, 5 = *Slightly agree*, 6 = *Moderately agree*, 7 = *Completely agree*)

TE1: In daily life, I often feel sympathy and concern for those who encounter misfortune. (1-7)

TE2: When I see others in urgent or sad situations, I am easily infected by their emotions. (1-7)

TE3: Even for fictional novel or movie plots, I can easily put myself into the characters’ feelings. (1-7)

**Part 3: Viewing situation arousal**

Instructions: Next, please carefully recall a fast-paced cutting-edge film or television work with a compact plot and strong conflicts that you have watched within the last week (or that left the deepest impression on you recently) (including theatrical movies, web series, micro-dramas, etc.). For all the following questions, please answer completely based on your actual experience with this specific work.

**2. The name (or abbreviation) of this film/television work (or micro-drama) you recalled is: ___________**

**3. The core genre of this work is (please select the most appropriate one):** [Single choice]

Suspense / Reasoning / Thriller / Crime

Comedy / Relaxing / Daily life

Romance / Sweet love / Urban

Action / Sci-Fi / Fantasy

History / Costume / Martial arts

Realistic theme / Family ethics

Other: ___________

**Part 4: Core variable measurement scales**

Instructions: Please rate the following descriptions based on your personal feelings when watching the work you filled in above.

(1 = Completely disagree, 2 = Moderately disagree, 3 = Slightly disagree, 4 = Neutral, 5 = Slightly agree, 6 = Moderately agree, 7 = Completely agree)

**A. Perceived Narrative Rhythm (PNR)**

PNR1: I feel that the story plot of this work advances very fast, without any dragging. (1-7)

PNR2: The contradictions and conflicts in the drama erupt densely, and the amount of information in each episode/segment is enormous. (1-7)

PNR3: The screen transitions and shot scheduling of this work give me a very compact and high-pressure visual impact. (1-7)

PNR4: Its plot does not just blindly pursue speed, but the climax and soothing stages alternate exquisitely, guiding me like breathing. (1-7)

PNR5: This narrative rhythm and information density force me to be fully concentrated to keep up with the plot. (1-7)

**B. Attentional Allocation (AA)**

AA1: When watching, my attention is completely absorbed onto the screen, and I rarely think about the trivial matters of real life. (1-7)

AA2: When watching, I hardly notice what is happening in the surrounding real environment (such as mobile phone pop-ups, movements around me). (1-7)

AA3: This high-intensity viewing experience makes me completely lose my perception of the passage of real time. (1-7)

AA4: When watching this work, it is actually easy for me to switch to a small window, look at other things, or get distracted. (1-7)

AA5: Even when encountering slightly gentle plot segments, it is hard for me to take my eyes off the screen. (1-7)

**C. Narrative Transportation (NT)**

NT1: As I keep watching, I feel as if I have crossed the boundary of the screen and entered the virtual world constructed by the story. (1-7)

NT2: The situations depicted in the drama allow me to immediately generate very vivid and fresh visual associations in my mind. (1-7)

NT3: To verify that you are reading the question stem carefully, please directly select the option labeled “1” for this question. (1-7)

NT4: I feel like an “invisible witness” who just happens to stand at the scene witnessing the events unfold, rather than an audience sitting outside the screen. (1-7)

NT5: When I am completely immersed in the plot, I even briefly forget the physical environment I am truly in. (1-7)

**D. Emotional Immersion (EI)**

EI1: The emotions of the characters in the drama deeply touch me (for example, when they are frustrated, I also feel genuinely sad or angry). (1-7)

EI2: When watching climactic segments, I experience actual physiological or emotional responses (such as accelerated heartbeat, holding my breath, or moist eyes). (1-7)

EI3: This work largely breaks through my psychological defense line and arouses strong personal emotional resonance. (1-7)

EI4: After watching (or when paused), the intense emotional atmosphere brought by the drama still continues to resonate in my heart for a long time. (1-7)

**Part 5: Demographics and confounding control variables**

**4. Your biological sex:**

Male

Female

**5. Your age group is:**

18 - 25 years old

26 - 35 years old

36 - 45 years old

46 years old and above

**6. Your highest level of education (including currently enrolled):**

High school and below

Associate degree

Bachelor’s degree

Master’s degree and above

**7. When watching the work you just filled in, which device screen did you mainly use?**

Vertical mobile phone screen (Common for micro-dramas/short videos)

Horizontal mobile phone screen / Tablet computer

Computer monitor / Laptop computer

TV / Projector / Cinema big screen

**8. On average, what is the total time you spend watching various film/television/micro-dramas every day?**

Less than 1 hour

1 - 2 hours

2 - 4 hours

More than 4 hours

**Appendix B: Data Codebook and Variable Value Matrix**

**Variable Overview and Value Matrix**

| Variable Code (Variable Code) | Variable Name and Item Definition (Item Description) | Data Value Description (Value Labels) | Variable Type (Data Type) |
| --- | --- | --- | --- |
| Consent | Informed consent and age screening. Are you at least 18 years old and voluntarily participating? | 1 = 18 years of age or older and agree to participate 2 = Under 18 years of age or do not agree | Categorical variable (Nominal) (for screening) |
| TE1 TE2 TE3 | Trait Empathy (Trait Empathy) TE1: Feel sympathy for those who suffer misfortune… TE2: Easily infected by others’ negative emotions… TE3: Easily substitute into fictional novel/movie characters… | 1 = Strongly Disagree (Strongly Disagree) … 4 = Neutral (Neutral) … 7 = Strongly Agree (Strongly Agree) | Continuous/Scale variable (Scale) |
| Recall | Viewing situation arousal. Name of the film/television/micro-drama recently watched by the respondent | Open text record (Requires manual checking and cleaning) | String (String) |
| Genre | Core genre type | 1=Suspense/Reasoning/Crime 2=Comedy/Daily life 3=Romance/Urban 4=Action/Sci-Fi/Fantasy 5=History/Costume 6=Realistic/Family 7=Other | Categorical variable (Nominal) |
| PNR1 PNR2 PNR3 PNR4 PNR5 | Perceived Narrative Rhythm (Perceived Narrative Rhythm) PNR1: Narrative speed is extremely fast… PNR2: Contradictions/events are dense… PNR3: Visual tension brought by the picture/shot… PNR4: Rhythm fluctuation of alternating climax and low tide… PNR5: Mental load forcing full concentration… | 1 = Strongly Disagree (Strongly Disagree) … 4 = Neutral (Neutral) … 7 = Strongly Agree (Strongly Agree) | Continuous/Scale variable (Scale) |
| AA1 AA2 AA3 AA4 ® AA5 | Attentional Allocation (Attentional Allocation) AA1: Focus completely absorbed on the screen… AA2: Almost block out physical environment movements… AA3: Lose perception of real time… AA4: Easily distracted, switch out to small window, etc… AA5: Hard to look away even during gentle segments… | 1 = Strongly Disagree (Strongly Disagree) … 4 = Neutral (Neutral) … 7 = Strongly Agree (Strongly Agree) | Continuous/Scale variable (Scale) Note: AA4 requires reverse scoring |
| NT1 NT2 NT3 © NT4 NT5 | Narrative Transportation (Narrative Transportation) NT1: Cross the screen into the virtual world… NT2: Mind immediately generates vivid imagery… NT3: Lie detector question (Please directly select “1”)… NT4: Feel like an invisible witness… NT5: Briefly forget the real physical environment… | 1 = Strongly Disagree (Strongly Disagree) … 4 = Neutral (Neutral) … 7 = Strongly Agree (Strongly Agree) | Continuous/Scale variable (Scale) Note: NT3 is a screening baseline |
| EI1 EI2 EI3 EI4 | Emotional Immersion (Emotional Immersion) EI1: Emotions of characters arouse resonance and touch… EI2: Climax segments bring physiological/emotional reactions… EI3: Break through defenses, arouse strong resonance… EI4: Emotional atmosphere continues to oscillate within after ending… | 1 = Strongly Disagree (Strongly Disagree) … 4 = Neutral (Neutral) … 7 = Strongly Agree (Strongly Agree) | Continuous/Scale variable (Scale) |
| Gender | Biological sex | 1 = Male (Male) 2 = Female (Female) | Dichotomous variable (Dichotomous) |
| Age | Age group | 1 = 18-25 years old 2 = 26-35 years old 3 = 36-45 years old 4 = 46 years old and above | Ordinal variable (Ordinal) |
| Edu | Highest education | 1 = High school and below 2 = Associate degree 3 = Bachelor’s degree 4 = Master’s degree and above | Ordinal variable (Ordinal) |
| Device | Main viewing device screen | 1 = Vertical mobile phone screen 2 = Horizontal mobile phone screen/tablet 3 = Computer monitor/laptop 4 = TV/projector/cinema | Categorical variable (Nominal) |
| Time | Average daily viewing time | 1 = Less than 1 hour 2 = 1-2 hours 3 = 2-4 hours 4 = More than 4 hours | Ordinal variable (Ordinal) |
